# Vasculitis in a Child With the Hyper-IgM Variant of Ataxia-Telangiectasia

**DOI:** 10.3389/fped.2019.00390

**Published:** 2019-10-24

**Authors:** Anna K. Meyer, Mindy Banks, Tibor Nadasdy, Jennifer J. Clark, Rui Zheng, Erwin W. Gelfand, Jordan K. Abbott

**Affiliations:** ^1^Immunodeficiency Diagnosis and Treatment Program, Department of Pediatrics, National Jewish Health, Denver, CO, United States; ^2^Rocky Mountain Hospital for Children, Denver, CO, United States; ^3^Department of Pathology, Ohio State University, Columbus, OH, United States

**Keywords:** ataxia-telangiectasia, primary immunodeficiency, vasculitis, hyper IgM, granuloma, tumor necrosis factor, CD21lo

## Abstract

A subset of patients with Ataxia-Telangiectasia (A-T) have dramatically reduced levels of IgG, IgA, and IgE with retained or elevated IgM levels. Several reports suggest that these A-T patients with a “hyper-IgM phenotype” (HIgM) suffer more clinical immunologic consequences than other A-T patients. The immunopathologic mechanism driving this phenomenon is unknown, making it difficult to predict response to immunomodulatory therapy. We describe an A-T patient with HIgM who underwent tumor necrosis factor (TNF) receptor blockade for cutaneous granuloma and after several months of successful therapy developed non-malignant lymphoproliferation, cytopenia, and increased serum immunoglobulin levels. This process was subsequently followed by an immune-complex-mediated intrarenal small vessel vasculitis that led to renal failure. The vasculitis was successfully treated with rituximab and corticosteroids. This case underscores the importance of HIgM as an unfavorable prognostic indicator in A-T and highlights the complexity of immunomodulatory treatment in this population, and the potential for a successful approach tailored to the immune defect.

## Introduction

The immunologic defects seen in Ataxia-Telangiectasia (A-T) are varied (1) and result in severe clinical morbidity in a subset of patients. One third of patients have no immunologic abnormality, whereas the remaining two-thirds have some combination of lymphopenia, immunoglobulin isotype or subclass deficiency, decreased naïve T cells, and abnormal vaccine responses (2). Despite laboratory evidence of immune abnormalities, most of these A-T patients suffer no more than repeated sinopulmonary infections without more serious infectious consequences (3). Approximately 10% of A-T patients exhibit a “hyper-IgM phenotype (HIgM),” with elevated IgM and severe deficiency of other immunoglobulin isotypes (4). Unlike the majority of A-T patients, HIgM A-T patients suffer potentially life threatening immune complications, such as autoimmunity and lymphoproliferative disease, and worse long term outcomes (5, 6).

Another minority of A-T patients develop severe cutaneous granulomas (7, 8). These lesions most frequently present as nodules on the face and extremities that can progress to large, ulcerating plaques (9). Their etiology is not well-defined, but reports have identified reduced naïve CD8 T cell and IgA levels within the blood of patients (8) and increased ratios of CD8 to CD4 T cells within the granulomas (10). More recently, many of these granulomas have been found to contain vaccine-strain rubella antigen (11). Treatment generally consists of corticosteroids, tumor necrosis factor (TNF) inhibition, or nitazoxanide with responses reported in the literature variable at best (8, 9, 12).

We report the clinical course of an A-T patient with HIgM in whom the TNF inhibitor, adalimumab, was used to treat chronic cutaneous granulomas. She experienced gradual improvement in the size and appearance of the granulomas; however, several months into her therapy, she developed severe immunologic dysregulation ultimately leading to immune-complex vasculitis and renal failure. Multiple immunomodulatory insults including anti-TNF therapy, chronic granuloma, and acute infection with norovirus may have contributed to the immune dysregulation seen in this patient. Nonetheless, the vasculitis was suppressed by a targeted approach, and the patient remains in remission.

## Case Report

**Figure 1:**
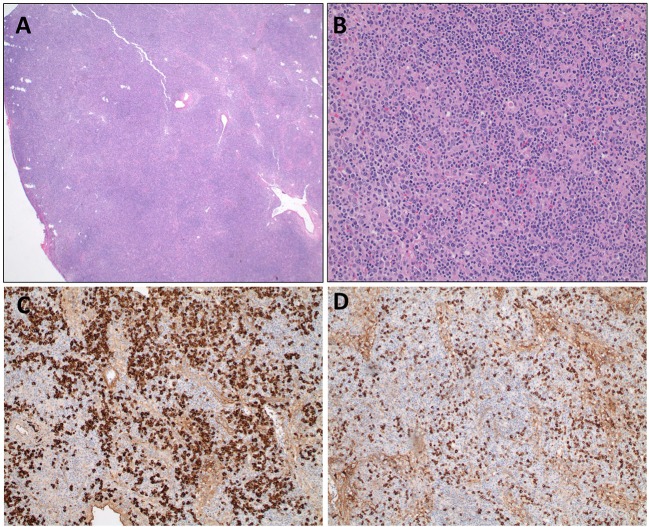
Axillary lymph node biopsy from an HIgM A-T patient. **(A)** Lymph node biopsy with paracortical expansion (**A**, 20X) consisting of a mixture of small lymphocytes, plasma cells, and immunoblasts (**B**, 200X). Immunohistochemical staining for IgD **(C)** and IgM **(D)** demonstrate cytoplasmic staining of plasma cells and lymphocytes.

**Figure 2:**
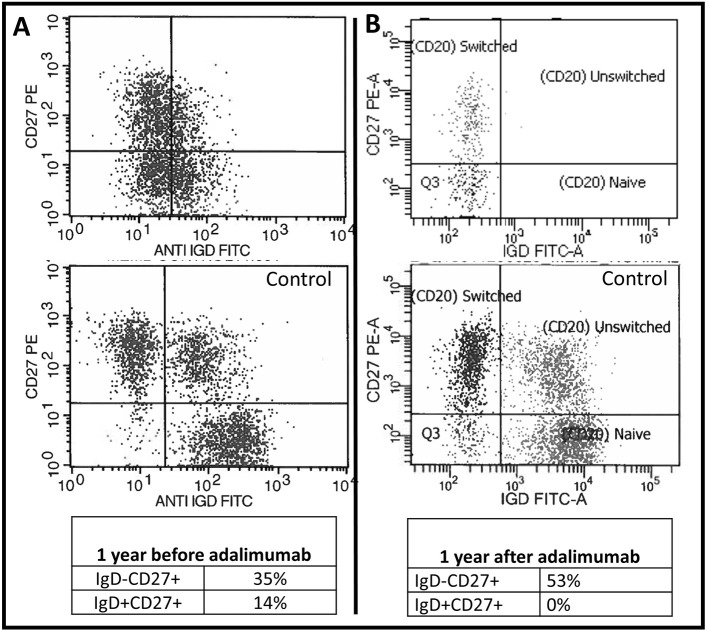
Baseline B cell abnormalities worsened following TNF blockade. Two-dimensional flow cytometry plot of the patient's CD20+ B cells 1 year before **(A)** and 1 year after **(B)** initiating adalimumab therapy. The patient's plots are on top and underneath each plot is the same day normal control sample.

We report the extended follow up of a 6-year-old girl with HIgM A-T and a history of rubella-positive cutaneous granuloma whose initial evaluation was reported previously (13). Her initial immunological findings included elevated serum IgM levels with absent IgA and IgG, increased percentage of CD19^+^CD38^lo^CD27^−^CD10^−^CD21^−/low^ B cells, increased percentage of circulating T follicular helper cells (Tfh cells), and decreased percentage and function of regulatory T cells. Due to progression of her cutaneous granulomas while on topical and systemic corticosteroid therapy, she started adalimumab10 mg subcutaneously every 2 weeks. She experienced partial remission of the lesions, but 1 year into her treatment, she developed diffuse lymphadenopathy, splenomegaly, and cytopenia, and adalimumab was discontinued. An axillary lymph node biopsy showed predominant paracortical hyperplasia, sparse residual germinal centers with follicular dendritic cell networks, collections of Tfh cells, and abundant polytypic, plasmablasts (Figures 1A,B). There were no granulomas, and EBV was not detected. IgM and IgD were detected diffusely (Figures 1C,D), IgG staining was sparse, and IgA staining was absent. Serum IgM level at the time of biopsy had increased to 4,000 mg/dl, and her blood CD20+ B cells experienced a global decrease in surface IgD expression (Figures 2A,B). Oral corticosteroid treatment was initiated, and the lymphadenopathy and splenomegaly demonstrated mild improvement.

**Figure 3:**
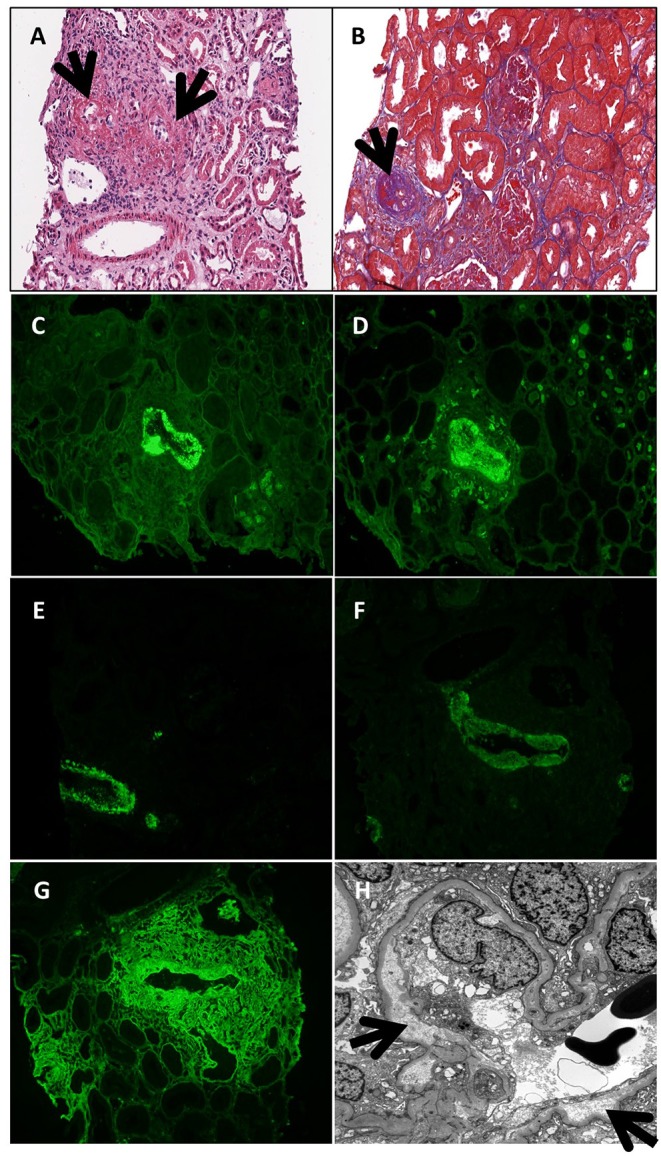
Renal biopsy at age 6 years. Necrotic and inflamed arteries (black arrows; **A**—hematoxylin eosin stain; **B**—trichrome stain). IgG **(C)**, IgM **(D)**, C3 **(E)**, C1q **(F)**, and fibrinogen **(G)** deposition seen in a small necrotic artery by immunofluorescent staining. **(H)** Electron micrograph demonstrating subendothelial widening along a glomerular capillary loop (black arrows).

Two months after the onset of lymphoproliferation, the patient was admitted with fever and diarrhea and subsequently developed acute kidney failure. Infectious workup identified norovirus in the stool but was otherwise negative. Nitazoxanide was initiated with little impact on the fevers, progressive renal failure, or the cutaneous granulomas. The diarrhea gradually resolved. She became oliguric at week 4 of admission with a serum creatinine peak of 2.2 mg/dL (Table 1). Renal biopsy was complicated by uncontrollable bleeding necessitating nephrectomy. Histologic staining of the kidney demonstrated acute and chronic necrotic, inflamed arteries with thrombosis and various amounts of intimal thickening (Figures 3A,B,G,H), and strong IgG, IgM, C3, and C1q staining (Figures 3C–F). Serum C3 and C4 levels were decreased (Table 1). Serum immunoglobulin levels were remarkable for an IgM level of 326 mg/dl and normal levels of IgA (63 mg/dl), a change from prior examinations when her IgM was over 4,000 mg/dl and her IgA was below the limits of detection. Four weekly doses of rituximab were administered in conjunction with pulse and maintenance methylprednisone. Over the next 2 weeks, complement levels normalized, IgA levels dropped below the assay limit of detection, and renal function returned to normal. Just prior to hospital discharge, she was transitioned to oral prednisolone, started on mycophenolate, and continued monthly IVIG. One year later, the vasculitis remains in remission, but the cutaneous granulomas have worsened and are now detected in her bone marrow.

**Table 1:** Results of immunologic studies and serum creatinine levels.

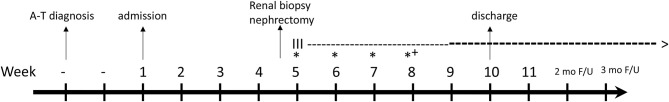																
																Normal Ranges
IgM (mg/dL)	719	>4,000					326		445	228	220		218			44–266 mg/dL
IgA (mg/dL)	<10	<40					63						<10	<10		43–253 mg/dL
C3 (mg/dL)				49			77	93		110	154		170	166	138	70–206 mg/dL
C4 (mg/dL)				<8						21.2	30.6		31.9	37.7	33.8	11–61 mg/dL
Cr (mg/dL)		0.5	1.2	1.7	1.2	2.2	0.6	0.5	3.0	0.9						0.3–1 mg/dL
ESR		99											28			0–20 mm/h
PLT	261	86	70										103			150–500 K/uL

*(^*^) rituximab 270 mg, (+) mycophenolate mofetil 300 mg twice daily, (I) high-dose methylprednisone, (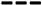) IV methylprednisone, (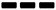) oral prednisone*.

## Discussion

The HIgM phenotype of A-T is associated with more severe immunologic disease, including autoimmunity and lymphoproliferative disease, and worse outcomes than the remainder of A-T patients (4, 5, 14–21). As illustrated in the presented case, the poorer clinical outcomes are often preceded by immune dysregulation and progress to autoimmune or inflammatory organ damage that can be fatal. While the reason for such severe clinical deterioration is poorly understood, it appears from this case that multiple factors contribute to clinical worsening. At baseline, the patient had lymphopenia and abnormal class switching. Superimposed on that background, she had vaccine-strain-rubella-containing cutaneous granulomas, a source of chronic immunologic stimulation. Her immune system was then subject to TNF blockade, a state that could have influenced both her baseline immunologic abnormalities and her response to chronic rubella antigen stimulation. Finally, she was infected with norovirus, an additional external stimulus too her immune system. It is likely that combination of these events set the stage for the immune-mediated vasculitis.

As noted previously (13), the patient had elevated numbers of Tfh, reduced Tregs, and an abundance of CD21lo B cells—all of which showed evidence of chronic IFNγ stimulation. This immunophenotypic pattern is frequently seen in rheumatic disease and complicated common variable immune deficiency (CVID) (22, 23); however, this pattern has not yet been studied in a large group of HIgM A-T patients. As a result, it is unknown whether this subgroup of A-T is inherently predisposed to this pattern of immune dysregulation, or if additional immunologic perturbations are required. Beyond the HIgM A-T phenotype, the discussed patient had a source of chronic antigen stimulation, rubella-associated cutaneous granulomatous disease. Tfh have been identified at the site of granuloma formation within mycobacteria-infected lung tissue (24), suggesting their role in controlling chronic intracellular infection. It is therefore worth asking if the expanded Tfh pattern as seen in this patient resulted from cutaneous granulomatous disease, HIgM phenotype, or the combination of the two? Additional research will be required to answer this question.

We also question whether the clinical developments were related to the institution of the anti-TNFα therapy used to control her progressive granulomatous disease? There are several reports of similar outcomes in patients treated with an anti-TNFα including a case of a patient with psoriasis who developed IgA glomerulonephritis after 18 months of TNF blockade with adalimumab (25, 26). Additionally, a small retrospective study of 8 patients reported development of cutaneous and systemic small-vessel vasculitis associated with the use of TNFα inhibitors (27). Unlike the previously reported anti-TNF-associated vasculitis patients who experienced a remission of vasculitis following anti-TNF discontinuation, this patient was off adalimumab for 2 months before the vasculitis developed. If TNF blockade played such a role in this patient, it would imply an irreversible change in the immune system, an adverse outcome that has not previously been described.

The mechanism that could explain such a relationship between TNF blockade and vasculitis is unclear. TNF-α is produced by activated B cells and supports their expansion in an autocrine manner (28), and there is evidence that T-cell-independent immunoglobulin production may be supported by TNF blockade (29–31). In addition, TNF inhibitors augment B-cell expression of TACI and TLR9, receptors capable of transmitting potent activation signals (32). Given these findings, it is conceivable that abnormal and possibly autoreactive B cell populations were somehow rescued by TNF inhibition in a T-cell-independent manner, particularly since the predominant B cell population was CD19^+^CD38^lo^CD27^−^CD10^−^CD21^−/low^, a subset typically enriched for autoreactive B cell receptors (33). However, in the context of autoimmune disease, rather than augmenting germinal center activity, blockade of TNF disrupts germinal center reactions and impairs T-cell-dependent vaccine responses (29, 34). The patient had a dramatic increase in Tfh cells, suggesting that T-cell help was an important component of her progressive autoimmunity. How TNF inhibition influences Tfh inside granulomas to our knowledge has not been explored. It seems unlikely that TNF blockade indirectly augments Tfh activity by generating more antigen to which T cells can react. In this case, adalimumab did not result in rubella dissemination, and in fact, the clinical appearance of the skin granulomas improved while on adalimumab. Therefore, while Tfh are implicated in granuloma formation and control of chronic infection, we do not conclude that TNF inhibition indirectly augmented Tfh activity by increasing availability of rubella antigens. It is also unlikely that TNF blockade directly affected the Tfh compartment, as it does not appear to be important for Tfh development (35). TNF inhibition is therefore difficult to implicate as the sole cause of Tfh expansion or Tfh-mediated autoimmunity in this case.

The immune dysregulation seen in the HIgM subset of A-T frequently leads to fatal outcomes. In this case, immune-mediated vasculitis was successfully treated with a combination of B-cell and T-cell targeted therapy. Rituximab was chosen because of the implication of autoantibodies in the disease process. The resulting elimination of an almost completely phenotypically abnormal B-cell compartment provided reassurance that ongoing autoantibody production was halted; however, the persistence of an expanded Tfh compartment required additional immunosuppression. Maintenance treatment with mycophenolate was prescribed at the time of discharge. She has not experienced relapse in the year following her discharge.

This case highlights the complexity of immune dysregulation and difficulty in utilizing immunomodulatory therapy in the HIgM subset of A-T. The complexity may be enhanced in the presence of chronic rubella-containing chronic granulomas in the face of dysregulation of both B and T cell compartments. The impact of immunomodulatory therapy and infection on these dysregulated immune cells can be difficult to predict, and patients should be monitored closely. Treatment of severe immune complications should be tailored to the clinical circumstance, where detailed immune system characterization can support designing specific treatment regimens.

## Concluding Remarks

This case highlights the importance of hyper IgM as an unfavorable prognostic indicator in A-T, explores the factors that preceded almost fatal severe immune disease, and describes the treatment approach that induced clinical remission.

## Data Availability Statement

The raw data supporting the conclusions of this manuscript will be made available by the authors, without undue reservation, to any qualified researcher.

## Ethics Statement

The subject of this case report was enrolled in a research study approved by the National Jewish Health Institutional Review Board. Written informed consent was obtained from the parents for the publication of any potentially identifiable images or data included in this article.

## Author Contributions

AM and JA co-wrote the manuscript. TN and RZ contributed pathological slides and edited the manuscript. JC, MB, and EG cared for the patient, discussed the manuscript, and edited the manuscript.

### Conflict of Interest

The authors declare that the research was conducted in the absence of any commercial or financial relationships that could be construed as a potential conflict of interest.
